# Red Light Regulates the Metabolite Biosynthesis in the Leaves of “Huangjinya” Through Amino Acid and Phenylpropanoid Metabolisms

**DOI:** 10.3389/fpls.2021.810888

**Published:** 2022-01-14

**Authors:** Qingping Ma, Laichao Song, Zhanhai Niu, Jingshan Li, Yu Wang, Haiwei Sun, Zhihong Ren, Hongxia Zhao, Shangjing Guo, Zhaotang Ding

**Affiliations:** ^1^College of Agronomy, Liaocheng University, Liaocheng, China; ^2^College of Horticulture, Qingdao Agricultural University, Qingdao, China; ^3^Taian Academy of Agricultural Sciences, Taian, China; ^4^Tea Research Institute, Shandong Academy of Agricultural Sciences, Rizhao, China

**Keywords:** *Camellia sinensis* cv. Huangjinya, transcriptome, red light, metabolome, glutathione *S*-transferase genes

## Abstract

“Huangjinya” is a light-sensitive albino variety and is widely cultivated in China. It has been proved that red light could promote the vegetable growth of plants. However, the mechanism of “Huangjinya” in response to a red light is unclear. This study used high-throughput sequencing technology to analyze the transcriptome of tender shoots of “Huangjinya” under the white and red light supplement conditions. At the same time, liquid chromatography tandem mass spectrometry (LC-MS) was used to analyze metabolite changes under different light conditions. Transcriptome analysis revealed that a total of 174 differentially expressed genes (DEGs) were identified after the red light supplement. Kyoto encyclopedia of genes and genomes (KEGG) classification indicated that amino acid metabolism enriched the most DEGs. In addition, two phenylpropanoid metabolism-related genes and five glutathione *S*-transferase genes (*CsGSTs*) were found to be expressed differently. Metabolome analysis revealed that 193 differential metabolites were obtained. Being the same as transcriptome analysis, most differential metabolites were enriched in amino acids, sweet and umami tasting amino acids were increased, and bitter-tasting amino acids were decreased after the red light supplement. In summary, red light supplementary treatment may be propitious to the quality of “Huangjinya” due to its regulatory effect on amino acid metabolism. Also, *CsGSTs* involved phenylpropanoid metabolism contributed to tea quality changes in “Huangjinya.”

## Introduction

Albino tea germplasm is one of the hot research topics in tea breeding. The phenotype and quality of albino tea cultivars were influenced by environmental factors, such as temperature and light (Feng et al., 2014; Song et al., 2017; Ma et al., 2018). Many studies were conducted to elucidate the potential molecular link between environment and albino phenotype or quality of tea plants (Chen et al., 2021, 2022; Yu et al., 2021).

“Huangjinya” (*Camellia sinensis* cv. Huangjinya) is a light-sensitive tea cultivar that shows yellow tender leaves under sunlight and would turn green irreversibly after shading. In yellow leaves of “Huangjinya,” the total chlorophyll and carotenoid contents were significantly lower than that in green leaves (Li et al., 2016). The isobaric tags for relative and absolute quantification (iTRAQ)-based proteome analysis revealed that the reduction of primary carbon metabolism and decrease in carbon skeleton leads to abnormal chloroplast development, then the chlorophyll and flavonoid biosynthesis was inhibited (Dong et al., 2018). As well, shading treatment mainly results in the changes in flavonoid, carotenoid, and amino acid biosynthesis-associated genes in “Huangjinya” (Song et al., 2017; Zhang et al., 2017). All of the above studies were about the effect of light intensity on “Huangjinya” by shading treatment. However, few studies were found about light quality on “Huangjinya.”

Light is an essential environmental factor for plant growth and plays important role in regulating plant growth and development. It affects plant growth through the changes in intensity and quality. For light quality, red light (650–760 nm) and blue light (430–470 nm) are major lights absorbed by chlorophyll. Both wavelengths of light control the photomorphogenesis in plants (De Wit et al., 2016). A lot of studies showed that red light mainly affects the vegetative growth of plants, such as promoting branch development, increasing leaf area, and improving plant fresh weight, while blue light mainly regulates plant reproductive growth, such as promoting flowering (Pedmale et al., 2016; Holalu and Finlayson, 2017; van Gelderen et al., 2018; Oh et al., 2020). In addition, red light could protect the plant against the damage by biotic and abiotic stresses (Ballare, 2014; Cao et al., 2018). Tender leaves were the major products of tea plants, so red light plays a more important role in “Huangjingya.” In addition, in the tender leaves of “Huangjinya,” chlorophyll *a* accounts for major proportion, which primarily absorbs red light (Song et al., 2017). Therefore, “Huangjinya” prefers red light for good growth and good quality. However, the molecular mechanism of red light in regulating the growth and quality of “Huangjinya” is still unknown.

In this study, to explore the mechanism of “Huangjinya” in response to red light, the transcriptome and metabolome analyses of “Huangjinya” under red light and white light were conducted. Different expression genes and metabolites related to red light were evaluated to elucidate the effect of red light on the quality change in “Huangjinya.” The results of this study will provide a reference for improving the cultivation method for light-sensitive tea cultivars.

## Materials and Methods

### Plant Materials and Treatment

Cuttings of “Huangjinya” plants aged 2 years were cultivated in the greenhouse at 25 ± 3^°^C for 12 h illumination with 75% humidity. Tea plants were treated with red/white light (2:3, 6,000 lx) for 10 days, and the plants exposed to only white light (8,000 lx) were used as the control plants. All the light was provided by LED lamps. The tender leaves from the top of the plants were harvested and stored at −80^°^C after being frozen in liquid nitrogen for RNA extraction and metabolome analysis. Three biological replicates were conducted in both control and red-light supplementary groups.

### RNA Extraction and Transcriptome Sequencing

The Plant Quick RNA Isolation Kit (Huayueyang Biotech, Beijing, China) was used to extract total RNA from tea leaves. The concentration and quality of RNA were determined using Agilent 2,100 Bioanalyzer (California, CA, United States) and Nano-Drop 2000 ultra-micro spectrophotometer (ThermoFisher, Waltham, MA, United States). Then, 1 μg total RNA was used to prepare the RNA-seq transcriptome library according to the manual of NEBNext Ultra™ RNA Library Prep Kit for Illumina (NEB, Massachusetts, MA, United States). To select cDNA fragments of preferentially 240 bp in length, the library fragments were purified using the AMPure XP system (Beckman Coulter, Beverly, United States). After PCR amplification and purification of the PCR products, the library quality was assessed using Agilent Bioanalyzer 2,100. After cluster generation using TruSeq PE Cluster Kit version 4-cBot-HS (Illumina, California, CA, United States), the library was sequenced on the Illumina 2,500 sequencer for paired-end sequencing to obtain raw reads.

### Quality Assessment and Differentially Expressed Genes Identification

The clean reads were produced after removing low-quality reads, adapter, and poly-N containing reads. Q30 was used for evaluating the quality of clean reads. The clean reads were mapped to the tea reference genome^1^ using HISAT2 software^2^ (Xia et al., 2020). Gene function annotation was conducted based on the following databases: NCBI non-redundant protein sequences (Nr), NCBI non-redundant nucleotide sequences (Nt), protein family (Pfam), Clusters of Orthologous Groups of proteins (KOG/COG), Swiss-Prot, kyoto encyclopedia of genes and genomes (KEGG) Ortholog database, and gene ontology (GO).

Fragments per kilobase of per million mapped reads (FPKM) was used to calculate the gene expression levels. The differentially expressed genes (DEGs) were screened using DESeq2 software^3^, and the DEGs were defined as |log2FoldChange| > 1.5, *P*-adjust < 0.01.

### Metabolome Analysis

The metabolome analysis was performed using Metware Biotech Inc. (Wuhan, China) according to the following procedure: 100 mg freeze-dried crushed powder was extracted overnight at 4°C with 0.6 ml 70% aqueous methanol. After centrifugation at 10,000 g for 10 min, the extracts were absorbed by CNWBOND Carbon-GCB SPE Cartridge (Anpel, Shanghai, China) and filtrated with 0.22 μm membranes. A total of 4 μl sample extracts were analyzed using the UPLC system (SHIMADZU, Japan) with Waters ACQUITY UPLC HSS T3 C18 column (1.8 μm, 2.1 mm × 100 mm). The mobile phases A and B were super-pure water (containing 0.04% acetic acid) and acetonitrile (containing 0.04% acetic acid), respectively. The gradient program was performed as follows: phase B was increased to 95 from 5% in 10 min and kept for 1 min, then was decreased to 5% immediately and balanced to 14 min. The speed of the mobile phase was 0.35 ml/min, and the temperature of the column was 40°C.

The metabolites were ionized by electrospray ionization (ESI). They were identified according to the secondary spectrum of fragment ions based on the Metware database. The quantification of identified metabolites was performed with multiple reaction monitoring of triple quadrupole mass spectrometry. Peak area was used for calculating the relative content of metabolites. |Fold change| > 1 and variable importance in projection (VIP) of orthogonal partial least squares discriminant analysis (OPLS-DA) > 1 were considered as differential metabolites. Enrichment and classification of metabolites were conducted based on the KEGG database.

### Quantitative Real-Time PCR Analysis

To verify the accuracy of the transcriptome data, 9 different genes were randomly selected for expression level verification. Primers were designed using AlleleID 6.0 software^4^ (Supplementary Table 1). The quantitative real-time PCR (qRT-PCR) was conducted on the Bio-Rad CFX96 system with the following procedure: 95°C for 30 s; 95°C for 5 s; and 60°C for 30 s for 40 cycles. The total of 25 μl reaction volume included 0.8 μl 10 μM primers, 50 ng cDNA, and 12.5 μl 2X SYBR Green Fast qPCR Mix (Biomarker, China). *CsGAPDH* was used as the reference gene. The relative expression of genes was calculated using the 2^–ΔΔCt^ method (Livak and Schmittgen, 2001). Statistical analysis was conducted using SPSS 23.0^5^ and Microsoft Excel 2016. One-way ANOVA was used for evaluating the difference between groups, and *P* < 0.05 was considered a significant difference.

### Sequence Alignment and Phylogenetic Tree Construction

Glutathione *S*-transferase (GST) putative proteins from the tea plant and *Arabidopsis* were used for the construction of a phylogenetic tree by Mega X using the neighbor-joining method with 500 bootstrap replications (Kumar et al., 2016). The phylogenetic tree was rooted by Zeta GSTs from *Arabidopsis*. The motifs of the CsGST sequences were predicted using Multiple Em for Motif Elicitation (MEME) version 4.11.2 online^6^.

## Results

### Phenotype Changes in “Huangjinya” Shoots

**FIGURE 1:**
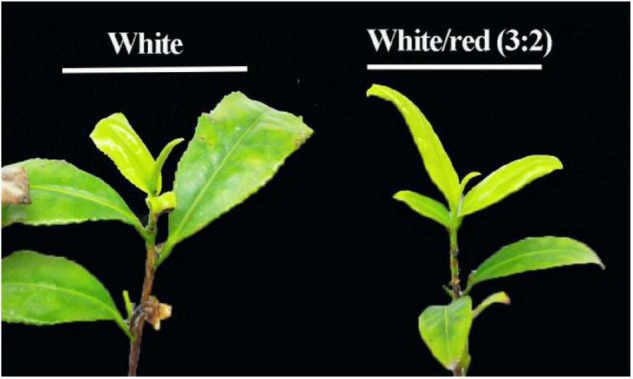
The phenotype of “Huangjinya” shoots under white and white/red (3:2) light conditions.

After the red light supplement, the color of tea shoots of “Huangjinya” was not changed significantly. However, the growth of tea shoots was quicker under the white/red (3:2) light than that under the white light condition. Therefore, the red light supplement could promote the growth of “Huangjinya” shoots (Figure 1).

### Quality Assessment of the RNA-Seq Data

A total of 57.29 Gb clean data was obtained through high-throughput sequencing (NCBI SRA accession: PRJNA774933). The Q30 of each sample was above 93.49%, which represents high quality. More than 86.76% of clean data were mapped to the genome with over 73.76% unique mapped reads (Table 1). These results indicated that the transcriptome data were enough for further identification of DEGs.

**TABLE 1:** Quality of the sequencing data.

Samples	Total reads	Clean reads	% ≥Q30	Mapped reads	Uniq mapped reads
HR-1	68,739,190	34,369,595	93.49%	86.76%	73.76%
HR-2	67,938,278	33,969,139	94.18%	87.31%	73.98%
HR-3	67,875,092	33,937,546	94.24%	86.94%	73.97%
HW-1	58,260,736	29,130,368	94.00%	86.92%	74.13%
HW-2	58,648,728	29,324,364	94.18%	87.11%	73.96%
HW-3	61,788,740	30,894,370	94.28%	87.18%	74.03%

*HR, tea leaves under red light; HW, tea leaves under white light.*

### Identification and Verification of Differentially Expressed Genes

**FIGURE 2:**
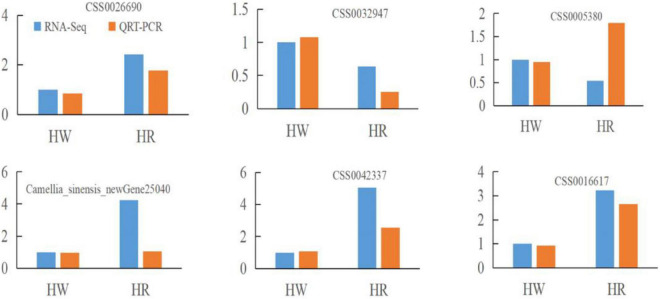
Quantitative real-time (qRT-PCR) verification for randomly selected differentially expressed genes (DEGs). Fold change of FPKM represents RNA-seq data. 2^–ΔΔCt^ represents qRT-PCR data.

After the red light supplement, 174 DEGs including 121 upregulated genes and 53 downregulated genes were identified. Of these, 165 DEGs were annotated in COG, GO, KEGG, KOG, NR, Pfam, and Swiss-Prot databases. To verify the reliability of the RNA-seq data of DEGs, 6 DEGs (CSS0026690, CSS0032947, CSS0005380, CSS0035186, CSS0042337, and CSS0016617) were randomly selected for the qRT-PCR analysis. The results showed similar trends in the expression of DEGs between RNA-seq and qRT-PCR, indicating that the RNA-seq analysis was reliable (Figure 2).

### Gene Ontology Classification of Differentially Expressed Genes

**FIGURE 3:**
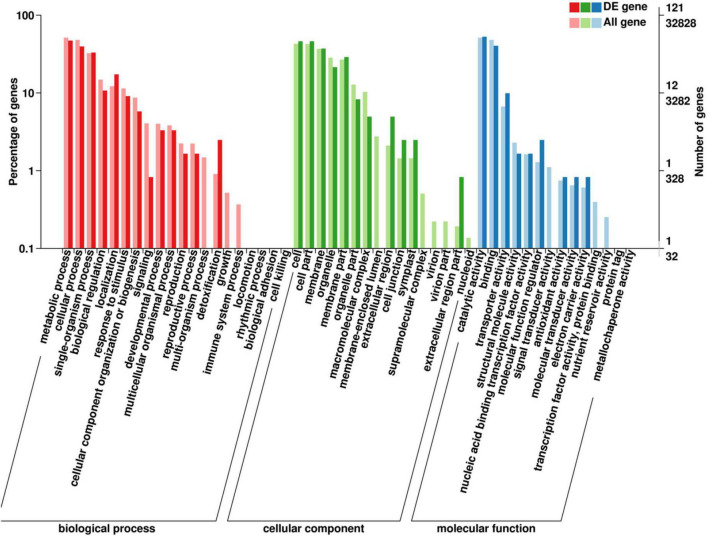
Gene ontology (GO) analysis of DEGs in “Huangjinya” after red light treatment. DE gene means a differentially expressed gene.

The GO classification indicated that detoxification of biological processes enriched a high proportion of DEGs (Figure 3). For cellular components, extracellular region, cell junction, and symplast enriched high percentages of DEGs. For molecular function, catalytic activity and binding enriched the most DEGs, and molecular function regulators enriched a high percentage of DEGs.

### COG Function Classification of Differentially Expressed Genes

**FIGURE 4:**
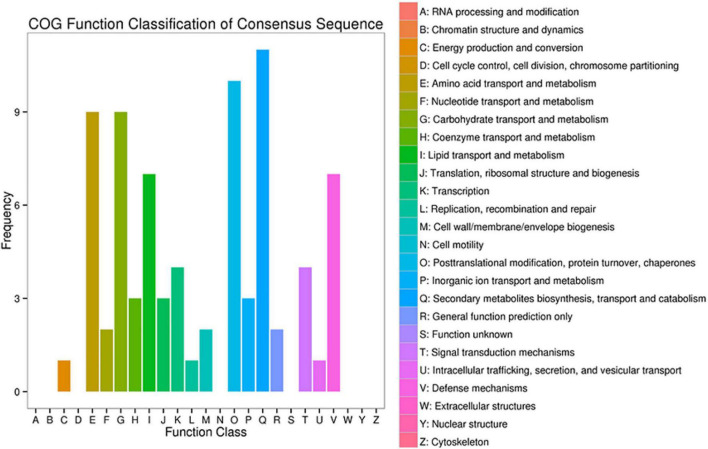
COG classification of DEGs in comparison to white and white/red (3:2) light conditions.

The COG function classification revealed that secondary metabolites biosynthesis enriched the most DEGs, followed by posttranslational modification, amino acid metabolism, and carbohydrate metabolism (Figure 4). Red light supplement caused changes in six amino acid transport and metabolism-related genes, including serine hydroxymethyltransferase (SHXMT, CSS0032127), cationic amino acid transporter 1 (CAAT1, CSS0029843, and CSS0020714), amino-acid acetyltransferase NAGS1 (CSS0050461), bifunctional methylthioribulose-1-phosphate dehydratase/enolase-phosphatase E1 (BMDE1, CSS0047160), and L-cysteine desulfhydrase (CDH, CSS0000409).

### KEGG Pathway Annotation of Differentially Expressed Genes

**FIGURE 5:**
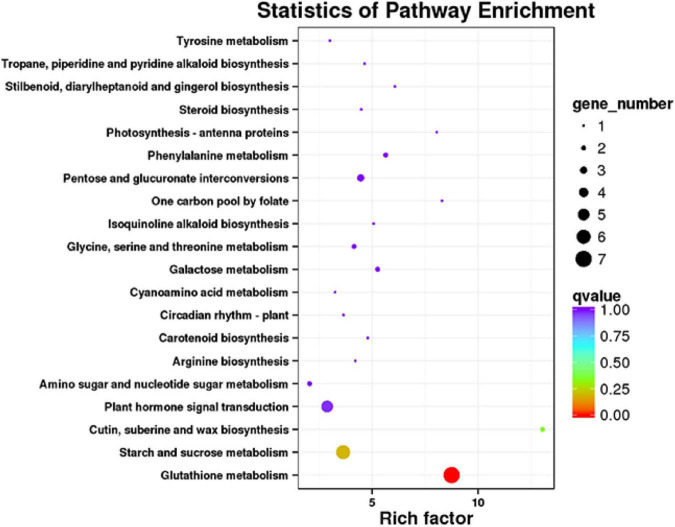
KEGG pathway enrichment analysis of DEGs. High rich factor and low *q*-value represent a high degree of enrichment.

There were 66 DEGs enriched in the KEGG pathways, and glutathione metabolism showed the highest rich factor and enriched the most DEGs, followed by starch and sucrose metabolism and plant hormone signal transduction (Figure 5). For plant hormone signal transduction, three-light signal transduction-related genes were enriched, including two-component response regulator ARR3 (CSS0036419), auxin transporter-like protein 3 (ATP3, CSS0016609), and auxin-responsive protein IAA26 (CSS0000357). These genes were upregulated after the red light supplement.

### Identification of Differential Metabolites

**FIGURE 6:**
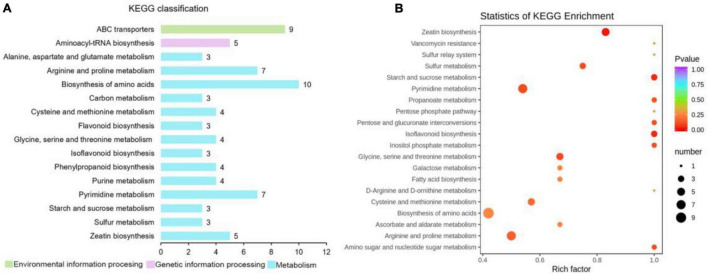
KEGG classification and pathway enrichment analysis of differential metabolites in “Huangjinya” tea shoots after red light supplement. **(A)** KEGG function classification of differential metabolites and **(B)** KEGG enrichment of differential metabolites.

A total of 523 metabolites were detected and identified in the leaves of “Huangjinya.” After the red light supplement, 193 differential metabolites were obtained, including 55 upregulated metabolites and 138 downregulated metabolites. KEGG annotation revealed that the biosynthesis of amino acids enriched the most differential metabolites (Figure 6 and Supplementary Table 2). Among the differential amino acids and derivatives, *N*-α-acetyl-L-glutamine and L-asparagine were increased after the red light supplement. Both metabolites were not only the source of umami taste but also the important amino acids for human health. In addition, L-histidine was increased, but L-arginine was decreased.

### Glutathione *S*-Transferase Genes in Response to Red Light

**FIGURE 7:**
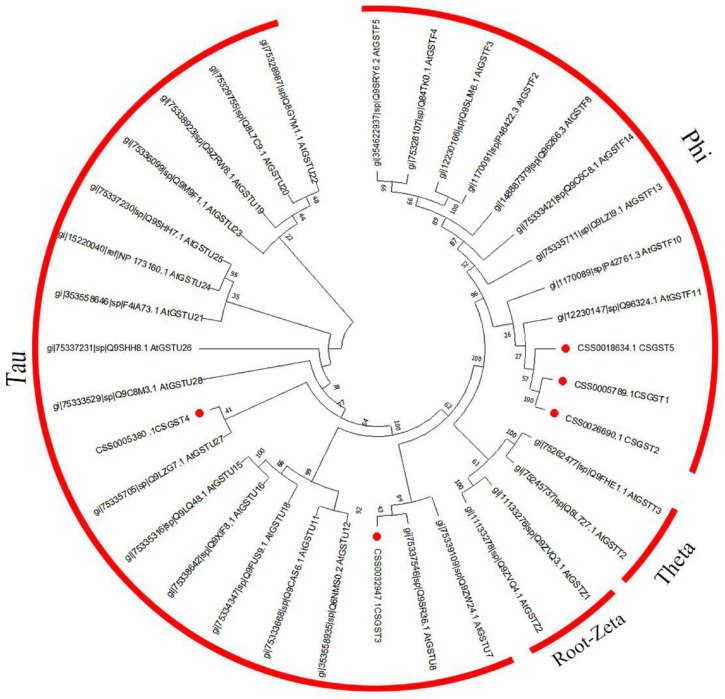
Phylogenetic tree of CsGSTs and AtGSTs from *Arabidopsis thaliana*.

**FIGURE 8:**
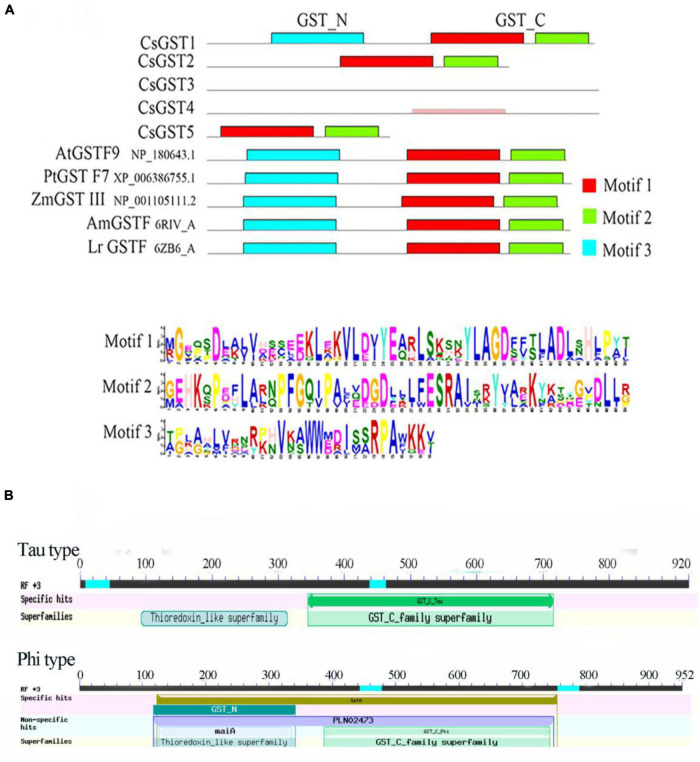
The protein structure analysis of CsGSTs. **(A)** The motif analysis of CsGSTs and **(B)** the conserved structure of CsGSTs.

According to the results of previous transcriptome sequencing, five glutathione *S*-transferase genes (CsGSTs) were differentially expressed, which were named CsGST1-5 (CSS0005789, CSS0026690, CSS0032947, CSS0005380, and CSS0018634). To classify these CsGSTs, a phylogenetic tree containing 30 AtGSTs from *Arabidopsis thaliana* and 5 CsGSTs was constructed. The results indicated that CsGST1, CsGST2, and CsGST5 were F-type GSTs, while CsGST3 and CsGST4 were U-type GSTs (Figure 7). Motif analysis revealed that F-type CsGSTs had conserved GSH-binding C-terminal domain (GST_C) and N-terminal domain (GST_N), which were the typical structures of the plant GST family (Kou et al., 2019; Yang et al., 2021). CsGST5 was shorter than CsGST1 and CsGST2, and only GST_N domain was identified in CsGST5 protein, suggesting that CsGST5 is a partial protein of the GST family (Figure 8A). Although no motif was found in CsGST3 and CsGST4, two conserved structures of GST-N-tau and GST-C-tau for tau type GST family were identified (Figure 8B), indicating that CsGST3 and CsGST4 were tau type GSTs.

### Glutathione *S*-Transferase GENES Involved Phenylpropanoid Metabolic Pathway

**FIGURE 9:**
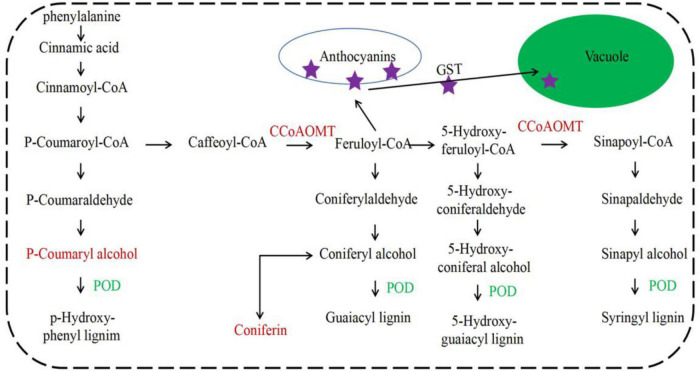
CsGSTs involved the phenylpropanoid metabolic pathway. CCoAOMT, caffeoyl-CoA *O*-methyltransferase; POD, peroxidase.

Plant glutathione *S*-transferase was mainly distributed in the cytoplasm, a few in chloroplasts and microbodies, and a small amount in the nucleus and apoplasts. The structure of the plant GSTs was highly conserved and had a similar three-dimensional structure (Nianiou-Obeidat et al., 2017). In the phenylpropanoid metabolism pathway, GST could transfer anthocyanins into the vacuole (Luo et al., 2018). Under red light treatment, the caffeoyl-CoA *O*-methyltransferase gene (CCoAOMT) was upregulated, and the peroxidase gene (POD) was downregulated. While *p*-coumaryl alcohol and coniferin were increased (Figure 9), *CCoAOMT* was the key gene in the upstream of anthocyanins biosynthesis. The upregulation of *CCoAOMT* and different expression of CsGSTs suggests that the biosynthesis and transport of anthocyanins by CsGSTs were accelerated.

## Discussion

In this study, the transcriptome and metabolome of the albino tea variety “Huangjinya” under different light conditions were analyzed to explore the effects of red light on the tea quality. A total of 174 DEGs were identified after the red light supplement, and amino acid metabolism enriched the most DEGs. Metabolome analysis revealed that *N*-α-acetyl-L-glutamine and L-asparagine were increased after the red light supplement. In addition, the phenylpropanoid metabolic pathway also enriched several DEGs and five *CsGSTs*, which contribute to anthocyanins, which were expressed differentially after the red light supplement.

Light is one of the most important environmental factors affecting photosynthesis and photomorphogenesis. In this study, three-light signal transduction-related genes were upregulated by red light supplement, including ARR3 and two auxin-responsive genes (ATP3 and IAA26). Among them, ARR3 was proved to be involved in the cross talk between light-induced phytochrome and cytokinin signal transduction (Grefen and Harter, 2004). Thus, it is suggested that red light could regulate the growth of “Huangjinya” shoots by activating the expression of auxin-responsive genes.

Amino acids are the important components deciding the tea quality. Each kind of amino acid has its unique taste, e.g., histidine, phenylalanine, and glycine taste sweet; glutamine tastes umami; and valine shows bitter taste (Bachmanov et al., 2016). In green tea, high umami and low bitter taste are desirable and usually represent high quality. Therefore, some albino tea cultivars with high amino acids, especially glutamine and theanine, were promoted. “Huangjinya” is an albino tea cultivar with high umami taste during the albino stage. However, its taste was unstable and affected by light. In this study, the red light induced the expression of amino acid metabolism-related genes, such as SHXMT, CAAT, and CDH. As well, two kinds of amino acids including *N*-α-acetyl-L-glutamine and L-asparagine were increased in “Huangjinya” after the red light supplement. Both amino acids contribute to the umami taste of tea. According to these results, the red light supplement could enhance the umami taste of tea by improving the corresponding amino acid contents. In addition, bitter-tasting amino acid L-arginine was decreased, and sweet-tasting amino acid L-histidine was increased. The changes in amino acids took a clue that the tea soup of “Huangjinya” was sweeter and umami but less bitter. This is a preferred taste of tea for most people. However, the relationships of identified DEGs in amino acid metabolism and the content changes of amino acids were still unclear. It needs plenty of studies to clarify these relationships.

Anthocyanin is an important composition of polyphenols and possesses antioxidant properties to protect humans from many chronic diseases by scavenging radicals and reducing reactive oxygen species formation (Bakuradze et al., 2019). Anthocyanin biosynthesis was associated with various environmental factors, such as temperature and light (An et al., 2020; Huyskens-Keil et al., 2020). In this study, *CCoAOMT*, which was a gene upstream of anthocyanin biosynthesis, was induced by red light. Meanwhile, *POD* in the lignin biosynthesis branch was suppressed. Lignin biosynthesis competed with anthocyanin biosynthesis in substrate consumption. Therefore, it suggests the promotion of anthocyanin biosynthesis. However, no differential anthocyanin components were identified after the red light supplement. It might be due to the upregulation of *CsGSTs*.

Under red light, five differentially expressed *CsGSTs* were detected, including 2 tau families and 3 phi families. They are the two major types of plant GSTs with the most members, and both types of GSTs have typical important functions such as detoxification and stress resistance (Yang et al., 2021). In addition, phi GSTs could transport flavonoid pigments to the vacuole (Hu et al., 2016; Kou et al., 2019). In tea plants, a combination of transcription factors *CsMYB75* and *CsGSTF1* could promote hyperaccumulation of anthocyanin (Wei et al., 2019). In this study, phi *CsGSTs* were upregulated under red light supplement, which might promote the transport of anthocyanins. However, the role of phi *CsGSTs* in red light-induced flavonoid biosynthesis in the shoots of “Huangjinya” needs detailed exploration.

## Conclusion

The red light supplement could promote the biosynthesis of umami and sweet amino acids but suppress the biosynthesis of bitter amino acids. Furthermore, anthocyanin biosynthesis and transport-related genes were induced by red light. Therefore, the red light supplement could improve the tea quality and taste of “Huangjinya.”

## Data Availability Statement

The raw data for RNA-Seq had been uploaded to NCBI SRA with accession of PRJNA774933. These data had been released on 2021-11-10.

## Author Contributions

QM, SG, and ZD conceived and designed the experiments. QM, ZN, JL, and ZR performed the experiments. LS, HS, and HZ conducted the data analysis. YW, SG, and ZD wrote and revised the manuscript. All authors contributed to the article and approved the submission.

## Conflict of Interest

The authors declare that the research was conducted in the absence of any commercial or financial relationships that could be construed as a potential conflict of interest.

## Publisher’s Note

All claims expressed in this article are solely those of the authors and do not necessarily represent those of their affiliated organizations, or those of the publisher, the editors and the reviewers. Any product that may be evaluated in this article, or claim that may be made by its manufacturer, is not guaranteed or endorsed by the publisher.

## Footnotes

1
http://tpdb.shengxin.ren/

2
https://daehwankimlab.github.io/hisat2/

3
https://www.bioconductor.org/packages/release/bioc/html/DESeq2.html

4
http://www.premierbiosoft.com/bacterial-identification/index.html

5
https://www.ibm.com/support/pages/spss-statistics-230-fix-pack-3

6
https://meme-suite.org/meme/tools/meme
