# Supplementary material for: Red Light Regulates the Metabolite Biosynthesis in the Leaves of “Huangjinya” Through Amino Acid and Phenylpropanoid Metabolisms
Source: Front Plant Sci. 2022 Jan 14;12:810888. doi: 10.3389/fpls.2021.810888 (PMC8797701; doi:10.3389/fpls.2021.810888)
Supplement: Supplementary file 1 [file Table_1.DOCX]

Table S1 Primers used for qRT-PCR.

| Gene ID | Length | Forward primer sequence (5′-3′) | Reverse primer sequence(5′-3′) |
| --- | --- | --- | --- |
| CSS0026690 | 180 | AAGAGAAAGCCCTGGTTGAC | ACCTCTGCTCATAGATGTCG |
| CSS0032947 | 171 | GGACTAAGTTCGCTGAGGAG | CCAAATACCCAATGCTTTCCC |
| CSS0005380 | 151 | TGTAGACGAAGAGAAAGAGGAAG | CTCATAGGACCAGCAGTATAAGG |
| CSS0035186 | 150 | GAAAGTATGCCGAGCGAGTG | CTGCTAACTGTATGTGCCTTGG |
| CSS0042337 | 200 | AACTGTTGCTGAGAAGGATG | GTATGACCACTTTGTTCTTAGC |
| CSS0016617 | 192 | TTTGAAGATCCAGACTTGTGTTC | TGTTTGCCGCTCTTGCTTAG |
| CsGAPDH  (XM_028237220) | 158 | TGGGTGTCAATGAGAAGGATTAC | TTTGTGTGGCTGTGATGGAG |
